# Supplementary material for: A Critical View of the Application of the APEX Software (Aqueous Photochemistry of Environmentally-Occurring Xenobiotics) to Predict Photoreaction Kinetics in Surface Freshwaters
Source: Molecules. 2019 Dec 18;25(1):9. doi: 10.3390/molecules25010009 (PMC7017383; doi:10.3390/molecules25010009)
Supplement: Supplementary file 1 [file molecules-25-00009-s001.zip › 3.2.4_gcc-4.4.0.zip!/3.2.4_gcc-4.4.0/3.2.4_gcc-4.4.0/doc/octave/html/interpreter/Simple-File-I_002fO.html]

Simple File I/O - Untitled


Next: Rational Approximations,
Previous: Terminal Input,
Up: Basic Input and Output


---

#### 14.1.3 Simple File I/O

The `save` and `load` commands allow data to be written to and
read from disk files in various formats. The default format of files
written by the `save` command can be controlled using the functions
`default_save_options` and `save_precision`.

As an example the following code creates a 3-by-3 matrix and saves it
to the file myfile.mat.

```
     A = [ 1:3; 4:6; 7:9 ];
     save myfile.mat A
```

Once one or more variables have been saved to a file, they can be
read into memory using the `load` command.

```
     load myfile.mat
     A
          -| A =
          -|
          -|    1   2   3
          -|    4   5   6
          -|    7   8   9
```

— Command: **save** file  
— Command: **save** options file  
— Command: **save** options file v1 v2 ...  
— Command: **save** options file -struct STRUCT f1 f2 ...  
> Save the named variables v1, v2, ..., in the file
> file. The special filename - may be used to write
> output to the terminal. If no variable names are listed, Octave saves
> all the variables in the current scope. Otherwise, full variable names or
> pattern syntax can be used to specify the variables to save.
> If the `-struct` modifier is used, fields f1 f2 ...
> of the scalar structure STRUCT are saved as if they were variables
> with corresponding names.
> Valid options for the `save` command are listed in the following table.
> Options that modify the output format override the format specified by
> `default_save_options`.
>
> If save is invoked using the functional form
>
> ```
>           save ("-option1", ..., "file", "v1", ...)
> ```
>
> then the options, file, and variable name arguments
> (v1, ...) must be specified as character strings.
>
> `-ascii`: Save a single matrix in a text file without header or any other information. `-binary`: Save the data in Octave's binary data format. `-float-binary`: Save the data in Octave's binary data format but only using single precision. Only use this format if you know that all the values to be saved can be represented in single precision. `-hdf5`: Save the data in HDF5 format. (HDF5 is a free, portable binary format developed by the National Center for Supercomputing Applications at the University of Illinois.) `-float-hdf5`: Save the data in HDF5 format but only using single precision. Only use this format if you know that all the values to be saved can be represented in single precision. `-V7` `-v7` `-7` `-mat7-binary`: Save the data in matlab's v7 binary data format. `-V6` `-v6` `-6` `-mat` `-mat-binary`: Save the data in matlab's v6 binary data format. `-V4` `-v4` `-4` `-mat4-binary`: Save the data in the binary format written by matlab version 4. `-text`: Save the data in Octave's text data format. (default). `-zip` `-z`: Use the gzip algorithm to compress the file. This works equally on files that are compressed with gzip outside of octave, and gzip can equally be used to convert the files for backward compatibility.
>
> The list of variables to save may use wildcard patterns containing
> the following special characters:
>
> `?`: Match any single character. `*`: Match zero or more characters. `[` list `]`: Match the list of characters specified by list. If the first character is `!` or `^`, match all characters except those specified by list. For example, the pattern `[a-zA-Z]` will match all lower and upper case alphabetic characters. Wildcards may also be used in the field name specifications when using the `-struct` modifier (but not in the struct name itself).
>
> Except when using the matlab binary data file format or the
> -ascii format, saving global
> variables also saves the global status of the variable. If the variable
> is restored at a later time using load, it will be restored as a
> global variable.
>
> The command
>
> ```
>           save -binary data a b*
> ```
>
> saves the variable a and all variables beginning with b to
> the file data in Octave's binary format.
>
>
>
> **See also:** load, default\_save\_options, dlmread, csvread, fread.

— Command: **load** file  
— Command: **load** options file  
— Command: **load** options file v1 v2 ...  
— Command: **S** = load("options", "file", "v1", "v2", ...)  
> Load the named variables v1, v2, ..., from the file
> file. If no variables are specified then all variables found in the
> file will be loaded. As with `save`, the list of variables to extract
> can be full names or use a pattern syntax. The format of the file is
> automatically detected but may be overridden by supplying the appropriate
> option.
>
> If load is invoked using the functional form
>
> ```
>           load ("-option1", ..., "file", "v1", ...)
> ```
>
> then the options, file, and variable name arguments
> (v1, ...) must be specified as character strings.
>
> If a variable that is not marked as global is loaded from a file when a
> global symbol with the same name already exists, it is loaded in the
> global symbol table. Also, if a variable is marked as global in a file
> and a local symbol exists, the local symbol is moved to the global
> symbol table and given the value from the file.
>
> If invoked with a single output argument, Octave returns data instead
> of inserting variables in the symbol table. If the data file contains
> only numbers (TAB- or space-delimited columns), a matrix of values is
> returned. Otherwise, `load` returns a structure with members
> corresponding to the names of the variables in the file.
>
> The `load` command can read data stored in Octave's text and
> binary formats, and matlab's binary format. If compiled with zlib
> support, it can also load gzip-compressed files. It will automatically
> detect the type of file and do conversion from different floating point
> formats (currently only IEEE big and little endian, though other formats
> may be added in the future).
>
> Valid options for `load` are listed in the following table.
>
> `-force`: This option is accepted for backward compatibility but is ignored. Octave now overwrites variables currently in memory with those of the same name found in the file. `-ascii`: Force Octave to assume the file contains columns of numbers in text format without any header or other information. Data in the file will be loaded as a single numeric matrix with the name of the variable derived from the name of the file. `-binary`: Force Octave to assume the file is in Octave's binary format. `-hdf5`: Force Octave to assume the file is in HDF5 format. (HDF5 is a free, portable binary format developed by the National Center for Supercomputing Applications at the University of Illinois.) Note that Octave can read HDF5 files not created by itself, but may skip some datasets in formats that it cannot support. `-import`: This option is accepted for backward compatibility but is ignored. Octave can now support multi-dimensional HDF data and automatically modifies variable names if they are invalid Octave identifiers. `-mat` `-mat-binary` `-6` `-v6` `-7` `-v7`: Force Octave to assume the file is in matlab's version 6 or 7 binary format. `-mat4-binary` `-4` `-v4` `-V4`: Force Octave to assume the file is in the binary format written by matlab version 4. `-text`: Force Octave to assume the file is in Octave's text format.
>
>
>
>
> **See also:** save, dlmwrite, csvwrite, fwrite.

There are three functions that modify the behavior of `save`.

— Built-in Function: val = **default\_save\_options** ()  
— Built-in Function: old\_val = **default\_save\_options** (new\_val)  
> Query or set the internal variable that specifies the default options
> for the `save` command, and defines the default format.
> Typical values include `"-ascii"`, `"-text -zip"`.
> The default value is `-text`.
>
>
>
> **See also:** save.

— Built-in Function: val = **save\_precision** ()  
— Built-in Function: old\_val = **save\_precision** (new\_val)  
> Query or set the internal variable that specifies the number of
> digits to keep when saving data in text format.

— Built-in Function: val = **save\_header\_format\_string** ()  
— Built-in Function: old\_val = **save\_header\_format\_string** (new\_val)  
> Query or set the internal variable that specifies the format
> string used for the comment line written at the beginning of
> text-format data files saved by Octave. The format string is
> passed to `strftime` and should begin with the character
> # and contain no newline characters. If the value of
> `save_header_format_string` is the empty string,
> the header comment is omitted from text-format data files. The
> default value is
>
> ```
>           "# Created by Octave VERSION, %a %b %d %H:%M:%S %Y %Z <USER@HOST>"
> ```
>
>
>
>
> **See also:** strftime, save.

— Built-in Function: **native\_float\_format** ()  
> Return the native floating point format as a string

It is possible to write data to a file in a similar way to the
`disp` function for writing data to the screen. The `fdisp`
works just like `disp` except its first argument is a file pointer
as created by `fopen`. As an example, the following code writes
to data myfile.txt.

```
     fid = fopen ("myfile.txt", "w");
     fdisp (fid, "3/8 is ");
     fdisp (fid, 3/8);
     fclose (fid);
```

See Opening and Closing Files, for details on how to use `fopen`
and `fclose`.

— Built-in Function: **fdisp** (fid, x)  
> Display the value of x on the stream fid. For example,
>
> ```
>           fdisp (stdout, "The value of pi is:"), fdisp (stdout, pi)
>           
>                -| the value of pi is:
>                -| 3.1416
> ```
>
> Note that the output from `fdisp` always ends with a newline.
>
>
>
> **See also:** disp.

Octave can also read and write matrices text files such as comma
separated lists.

— Function File: **dlmwrite** (file, a)  
— Function File: **dlmwrite** (file, a, delim, r, c)  
— Function File: **dlmwrite** (file, a, key, val ...)  
— Function File: **dlmwrite** (file, a, "-append", ...)  
> Write the matrix a to the named file using delimiters.
>
> The parameter delim specifies the delimiter to use to separate
> values on a row.
>
> The value of r specifies the number of delimiter-only lines to
> add to the start of the file.
>
> The value of c specifies the number of delimiters to prepend to
> each line of data.
>
> If the argument `"-append"` is given, append to the end of the
> file.
>
> In addition, the following keyword value pairs may appear at the end
> of the argument list:
>
> `"append"`: Either "on" or "off". See "-append" above. `"delimiter"`: See delim above. `"newline"`: The character(s) to use to separate each row. Three special cases exist for this option. "unix" is changed into '\n', "pc" is changed into '\r\n', and "mac" is changed into '\r'. Other values for this option are kept as is. `"roffset"`: See r above. `"coffset"`: See c above. `"precision"`: The precision to use when writing the file. It can either be a format string (as used by fprintf) or a number of significant digits.
>
> ```
>           dlmwrite ("file.csv", reshape (1:16, 4, 4));
> ```
>
> ```
>           dlmwrite ("file.tex", a, "delimiter", "&", "newline", "\\n")
> ```
>
>
>
>
> **See also:** dlmread, csvread, csvwrite.

— Loadable Function: data = **dlmread** (file)  
— Loadable Function: data = **dlmread** (file, sep)  
— Loadable Function: data = **dlmread** (file, sep, r0, c0)  
— Loadable Function: data = **dlmread** (file, sep, range)  
> Read the matrix data from a text file. If not defined the separator
> between fields is determined from the file itself. Otherwise the
> separation character is defined by sep.
>
> Given two scalar arguments r0 and c0, these define the starting
> row and column of the data to be read. These values are indexed from zero,
> such that the first row corresponds to an index of zero.
>
> The range parameter must be a 4 element vector containing the upper
> left and lower right corner `[`R0`,`C0`,`R1`,`C1`]` or
> a spreadsheet style range such as 'A2..Q15'. The lowest index value is zero.

— Function File: x = **csvwrite** (filename, x)  
> Write the matrix x to a file.
>
> This function is equivalent to
>
> ```
>           dlmwrite (filename, x, ",", ...)
> ```
>
>
>
>
> **See also:** dlmread, dlmwrite, csvread.

— Function File: x = **csvread** (filename)  
> Read the matrix x from a file.
>
> This function is equivalent to
>
> ```
>           dlmread (filename, "," , ...)
> ```
>
>
>
>
> **See also:** dlmread, dlmwrite, csvwrite.

- Saving Data on Unexpected Exits
